# Macrophages confer resistance to PI3K inhibitor GDC-0941 in breast cancer through the activation of NF-κB signaling

**DOI:** 10.1038/s41419-018-0849-6

**Published:** 2018-07-24

**Authors:** Muhammad Waqas Usman, Jing Gao, Tiezheng Zheng, Chunhua Rui, Ting Li, Xing Bian, Hailing Cheng, Pixu Liu, Fuwen Luo

**Affiliations:** 1 Cancer Institute, Department of Acute Abdomen Surgery, The Second Hospital of Dalian Medical University, Institute of Cancer Stem Cell, Dalian Medical University, Dalian, 116044 China; 2 Department of Physiology, Institute of Basic Medical Sciences, Dalian Medical University, Dalian, 116044 China; 3 College of Pharmacy, Dalian Medical University, Dalian, 116044 China

## Abstract

The PI3K pathway is one of the most dysregulated signaling pathways in epithelial cancers and has become an attractive therapeutic target under active preclinical and clinical development. However, recent clinical trial studies revealed that blockade of PI3K activity in advanced cancer often leads to the development of resistance and relapse of the diseases. Intense efforts have been made to elucidate resistance mechanisms and identify rational drug combinations with PI3K inhibitors in solid tumors. In the current study, we found that PI3K inhibition by GDC-0941 increased macrophage infiltration and induced the expression of macrophage-associated cytokines and chemokines in the mouse 4T1 breast tumor model. Using the in vitro co-culture system, we showed that the presence of macrophages led to the activation of NF-κB signaling in 4T1 tumor cells, rendering tumor cells resistant to PI3K inhibition by GDC-0941. Furthermore, we found that Aspirin could block the activation of NF-κB signaling induced by PI3K inhibition, and combined use of GDC-0941 and Aspirin resulted in attenuated cell growth and enhanced apoptosis of 4T1 cells in the in vitro co-culture system with the presence of macrophages. Consistently, the combination treatment also effectively reduced tumor burden, macrophage infiltration and pulmonary metastasis in in vivo 4T1 breast tumor model. Together, our results suggested macrophages in microenvironment may contribute to the resistance of breast cancer cells to PI3K inhibition and reveal a new combination paradigm to improve the efficacy of PI3K-targeted therapy.

## Introduction

PI3Ks (phosphatidylinositol 3 kinases) play an important role in many cellular processes, including cell proliferation, survival, and metabolism. The PI3K pathway is one of the most frequently altered signaling pathways in human cancer, including breast cancer^1,2^. Intense preclinical and clinical efforts have been made to develop effective PI3K-targeted therapies. However, responses of solid tumors to PI3K inhibitor monotherapy have been modest and often accompanied by rapid emergence of drug resistance^2,3^. There is thus an urging need to identify resistance mechanisms and develop rational combination therapies that will overcome the drug resistance.

Although significant efforts on PI3K signaling have been focused on feedback regulation and crosstalk with receptor tyrosine kinases and other signaling pathways^3–5^, recent findings revealed novel roles of the tumor microenvironment (extrinsic mechanism) in regulating therapeutic response and resistance^6,7^. Gene-expression analyses showed that increased gene signature of tumor microenvironment predicts resistance to neoadjuvant chemotherapy in estrogen-negative breast cancer^8^. In addition, as an important component of tumor microenvironment, tumor-associated macrophages (TAMs) induce chemotherapy resistance through secreting survival factors and/or activating anti-apoptotic signaling pathways in cancer cells^9^. In several solid tumor types including breast cancer, high densities of TAMs have been found associated with poor clinical outcomes^10^. Thus, blocking the recruitment, survival, and tumor-promoting activity of TAMs may present a promising strategy to overcome the resistance to PI3K inhibitors in solid tumors.

The IKK/nuclear factor-κB (NF-κB) pathway plays an important role in diverse cellular functions, including cell proliferation, survival, and inflammation^11,12^. The NF-κB signaling pathway is frequently hyperactivated in many tumor types including breast cancer^13,14^. Inactivation of the NF-κB pathway by knocking down *TNFα* in breast cancer led to suppressed cell proliferation and enhanced apoptosis^15^. Conditional deletion of *IKKb* in mouse melanocytes resulted in attenuated NF-κB signaling and protected mice from developing oncogenic Hras-induced melanoma^16^. Nevertheless, the effect of NF-κB signaling on therapeutic response to PI3K inhibition remains elusive in breast cancer.

The current study aimed to search for mechanisms of resistance to PI3K inhibition as monotherapy. We found that PI3K inhibition by GDC-0941 resulted in increased number of macrophages (Mφ) and induced expression of several macrophage-associated cytokines and chemokines in the mouse 4T1 breast tumor model. We investigated whether macrophages could confer resistance to PI3K inhibition through the activation of the NF-κB signaling in 4T1 tumor cells. We also examined whether the addition of Aspirin, a non-steroidal anti-inflammatory drug, could improve the efficacy of PI3K inhibitor GDC-0941 through suppressing the NF-κB signaling in both in vitro co-culture system as well as in vivo 4T1 tumor model.

## Results

### 4T1 breast tumors showed resistance to PI3K inhibitor with enhanced macrophage infiltration in vivo

**Fig. 1:**
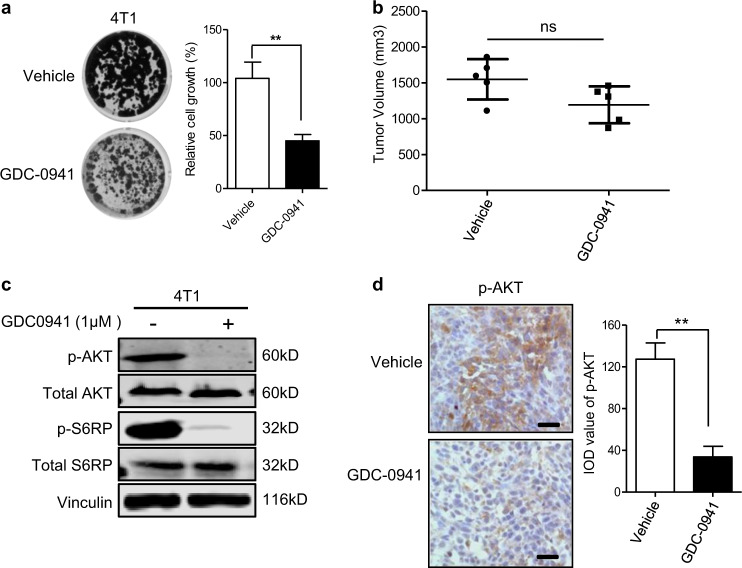
The growth inhibitory effects of GDC-0941 for 4T1 tumor cells in in vitro and in vivo assays were not consistant. **a** Clonogenic proliferation assay of 4T1 cells treated with 1 µM GDC-0941 or vehicle for 10 days. Means ± S.D. of three independent experiments are shown. **b** Tumor volumes in 4T1 allograft-bearing mice following treatment with GDC-0941 (125 mg/kg/day, oral gavage, *n* = 5) or vehicle control (*n* = 5) for 14 days. Error bars represent mean ± S.E.M. **c** Western blot analysis of proteins as indicated in 4T1 cells treated with 1 µM GDC-0941 or vehicle for 24 h. Vinculin was used as a loading control. **d** Representative images of immunohistochemical staining for p-AKT in tumors from mice bearing 4T1 allografts following treatment with GDC-0941 (*n* = 5) or vehicle control (*n* = 5) for 3 days. Scale bar, 50 μm. Quantification of the IOD value is shown. Error bars represent mean ± S.E.M. **P < 0.01 (Student’s *t*-test)

**Fig. 2:**
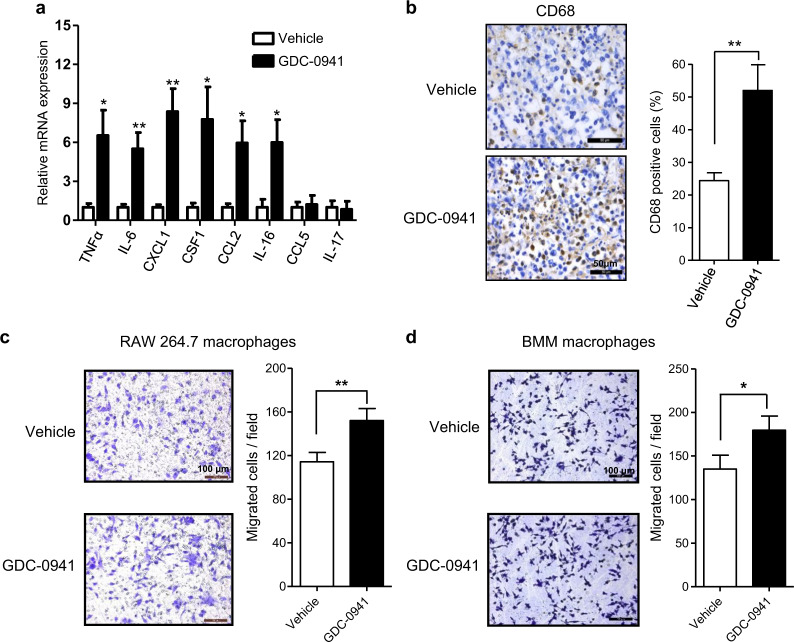
GDC-0941 treatment enhanced macrophage infiltration and provoked pro-inflammatory molecules in 4T1 allografts. **a** Quantitative RT-PCR analysis of a panel of cytokines and chemokines in 4T1 tumors of Balb/c mice following treatment with GDC-0941 (*n* = 5) or vehicle control (*n* = 5) for 3 days. Gene expression was normalized to β-actin. Error bars represent mean ± S.E.M. **b** Representative images of immunohistochemical staining for CD68 of 4T1 allografts after treatment with GDC-0941 or vehicle (*n* = 5 per treatment group) for 10 days. Scale bar, 50 μm. Percentages of positive cells of CD68 are shown. Error bars represent mean ± S.E.M. **c**, **d** Migration assay of RAW264.7 macrophages (**c**) and BMM macrophages (**d**) stimulated by conditioned media from 4T1 cells treated with 1 µM GDC-0941 or vehicle. Scale bars, 100 μm. Quantifications of migrated cells from three independent experiments are shown as mean ± S.D. *P < 0.05; **P < 0.01; ***P < 0.001 (Student’s *t*-test)

To investigate the effect of PI3K-targeted therapy on breast cancer, we subjected the 4T1 mouse breast tumor cells to PI3K inhibition by GDC-0941 both in vitro and in vivo. Interestingly, while the PI3K inhibitor GDC-0941 showed a significant suppressing effect on 4T1 cell proliferation in vitro (Fig. 1a), it only moderately slowed down the growth of 4T1 tumors established in the Balb/c mice (Fig. 1b). Of note, GDC-0941 treatment led to effective target inhibition of PI3K as evidenced by markedly reduced phosphorylated Akt signals both in vitro and in vivo (Fig. 1c, d). The discrepancy in the growth inhibitory effect of GDC-0941 in in vitro and in vivo assays prompted us to investigate whether tumor microenvironment may confer resistance to PI3K inhibitor GDC-0941 in vivo. For this, we first assessed the expression levels of a panel of cytokines and chemokines with reported links to microenvironment in tumor samples^9,17,18^. When compared to the vehicle group, GDC-0941-treated tumors exhibited a significant increase in mRNA expression of a panel of inflammatory cytokines/chemokines, including tumor necrosis factor α (TNFα), interleukin-6 (IL-6), colony-stimulating factor-1 (CSF1), chemokine (C-C motif) ligand 2 (CCL2), chemokine (C-X-C motif) ligand 1 (CXCL1), and IL-16 (Fig. 2a). We further confirmed an increase in the production of TNFα by enzyme-linked immunosorbent assay (ELISA) analysis and that of CSF1, CXCL1, and CCL2 by cytokine array analysis (Supplementary Figs. S1 and S2). Of note, all of these cytokines/chemokines were previously shown to be associated with macrophages^9^, suggesting a potential involvement of macrophages in the tumor response to PI3K inhibitor GDC-0941. Furthermore, tumor tissues from GDC-0941-treated mice also displayed significantly higher levels of CD68^+^ staining than those from the vehicle-treated group, suggesting that PI3K inhibition may induce macrophage infiltration (Fig. 2b). To look into this, we next examined the drug effect on the migration capability of macrophages. Both mouse leukemic RAW264.7 macrophages and freshly isolated mouse bone marrow-derived macrophages (BMMs) exhibited enhanced migration potential in the presence of conditioned media collected from GDC-0941-treated 4T1 cell culture (Fig. 2c, d). Taken together, these findings suggested that macrophage recruitment as a result of PI3K inhibition may contribute to the resistance of 4T1 tumors to GDC-0941 in vivo.

### Macrophages contributed to PI3K inhibitor resistance in the co-culture system with 4T1 cells

**Fig. 3:**
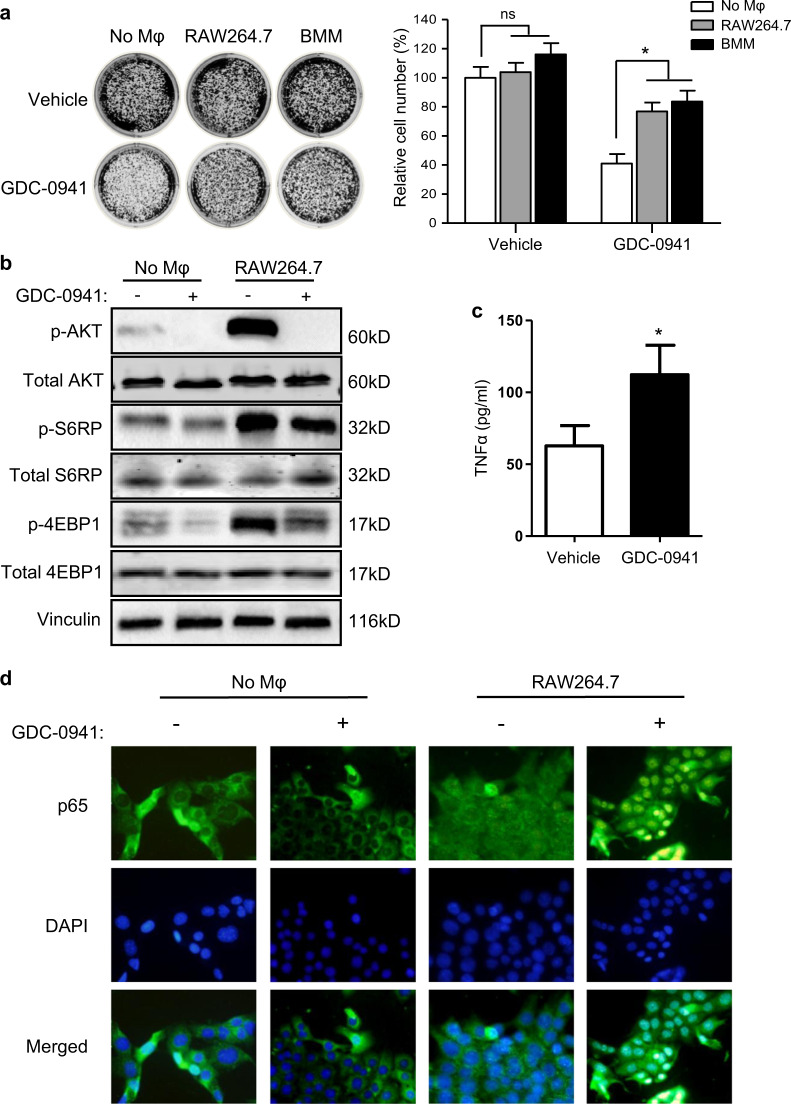
NF-κB signaling contributed to compromised efficacy of GDC-0941 in 4T1 cells in the presence of macrophages. **a** Clonogenic proliferation assay of 4T1 cells treated with 1 µM GDC-0941 or DMSO vehicle for 10 days. 4T1 cells were cultured alone or co-cultured with RAW264.7 or BMM macrophages as indicated. All experiments were performed in triplicate and representative images of plates are shown. Error bars represent mean ± S.D. **b** Western blot analysis of proteins as indicated in 4T1 cells treated with 1 µM GDC-0941 or vehicle for 24 h. 4T1 cells were cultured alone or co-cultured with RAW264.7 macrophages. Vinculin was used as a loading control. **c** ELISA analysis of TNFα in culture media from co-cultured 4T1 and RAW264.7 cells treated with 1 µM GDC-0941 or vehicle for 24 h. Means ± S.D. of three independent experiments are shown. **d** Representative images of immunofluorescent staining of p65 cellular localization in 4T1 cells treated with 1 µM GDC-0941 or vehicle for 24 h. 4T1 cells were cultured alone or co-cultured with RAW264.7 macrophages. Representative images of three independent experiments are shown. *P *<* 0.05; ***P < 0.001 (Student’s *t*-test)

Extensive evidence suggests that the immune microenvironment, especially macrophage, plays critical roles in promoting tumor growth and conferring resistance to targeted therapies^9,18^. To examine whether macrophages confer resistance to PI3K inhibitor GDC-0941 in 4T1 breast cancer cells, we co-cultured these cells with murine BMMs or RAW264.7 macrophages in a transwell system. GDC-0941 exhibited significantly less growth inhibitory effect on 4T1 cells co-cultured with BMMs or RAW264.7 macrophages than that on 4T1 cells cultured alone as assessed by clonogenic assays (Fig. 3a). Thus, macrophages may contribute to the resistance of 4T1 cells to PI3K inhibition in the co-culture system. Consistently, when compared to monoculture, 4T1 cells in the co-culture system with RAW264.7 macrophages displayed substantially increased abundance of AKT, S6RP, and 4EBP1 phosphorylated proteins, all of which are key components of the pro-survival PI3K/AKT/mTOR signaling pathway (Fig. 3b). While GDC-0941 treatment led to nearly completely abolished phosphorylated AKT signals in both culture systems, it had only a moderate inhibitory effect on phosphorylated S6RP and 4EBP1 signals in 4T1 cells co-cultured with macrophages (Fig. 3b). We reasoned that the remaining mTOR effector signaling may, at least in part, contribute to PI3K inhibitor resistance. Notably, consistent with enhanced recruitment of tumor-associated macrophages (TAMs) to tumors observed in vivo (Fig. 2b), ELISA analysis revealed a significant increase in the production of TNFα in the co-culture system in the presence of GDC-0941 when compared to the vehicle control. Together, these data suggest an involvement of macrophage-associated cytokines/chemokines in the mechanism of resistance to PI3K inhibition by GDC-0941.

As a pro-inflammatory cytokine, TNFα has been shown to promote proliferation and survival through activation of the NF-κB signaling pathway^19,20^. We next assessed the impact of PI3K inhibition on NF-κB signaling in 4T1 cells co-cultured with macrophages. Immunofluorescent staining analysis revealed that compared to 4T1 monoculture, the presence of macrophages rendered p65 localization in both nucleus and cytoplasm (Fig. 3d), suggesting macrophages may promote the activation of NF-κB signaling in 4T1 breast cancer cells. Moreover, GDC-0941 treatment resulted in an exclusive nuclear localization of p65 in 4T1 cells co-cultured with macrophages, indicative of NF-κB activation in response to PI3K inhibition (Fig. 3d). Together, these results suggest that macrophages in the tumor microenvironment may confer resistance to PI3K targeted therapy through inducing NF-κB activation.

### Aspirin augmented the sensitivity of breast cancer cells to PI3K inhibitor GDC-0941 through suppression of NF-κB signaling

**Fig. 4:**
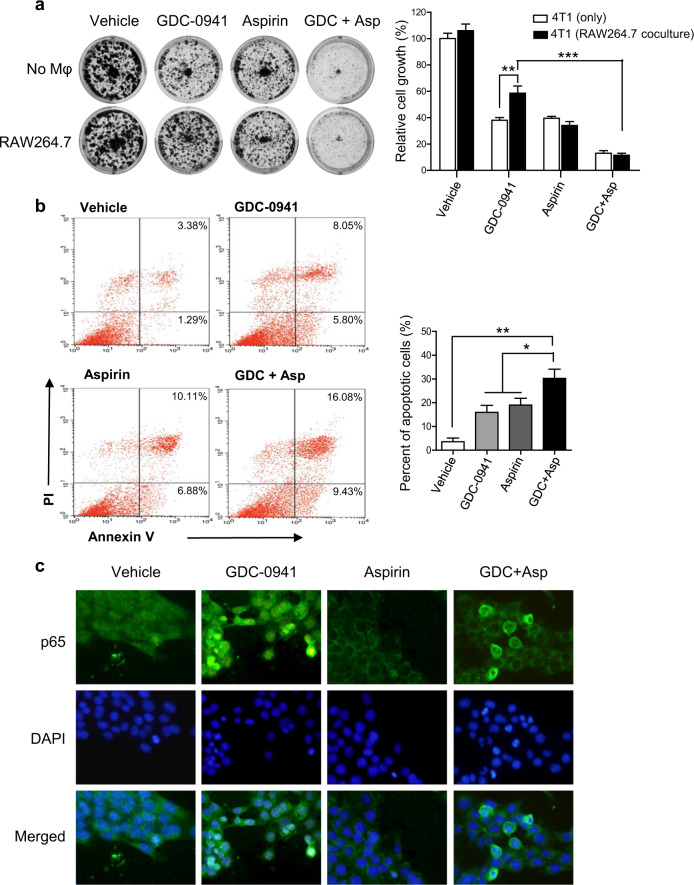
Aspirin inhibited the NF-κB signaling and enhanced the efficacy of GDC-0941 against 4T1 cells. **a** Clonogenic proliferation assay of 4T1 cells treated with 1 µM GDC-0941 and 3 mM Aspirin as single agents or in combination for 10 days. 4T1 cells were cultured alone or co-cultured with RAW276.7 macrophages. All experiments were performed in triplicate and representative images of plates are shown. Error bars represent mean ± S.D. **b** 4T1 cells treated with GDC-0941 and Aspirin as single agents or in combination for 48 h when co-cultured with RAW276.7 macrophages. The percentage of apoptotic cells was determined by Annexin V and PI staining. Means ± S.D. of three independent experiments are shown. **c** Representative images of immunofluorescent staining of p65 cellular localization in 4T1 cells treated as indicated for 24 h. 4T1 cells were co-cultured with RAW276.7 macrophages. Representative images of three independent experiments are shown. *P < 0.05; **P < 0.01; ***P < 0.001 (Student’s *t*-test)

It has been shown that the non-steroidal anti-inflammatory drug Aspirin is able to inhibit NF-κB activation^21–24^. We next used Aspirin to determine if suppression of NF-κB signaling may overcome resistance to PI3K inhibition in 4T1 cells co-cultured with macrophages. We found that Aspirin treatment alone attenuated the growth of 4T1 cells in both monoculture and co-culture systems, to a similar degree (Fig. 4a). Indeed, additional use of Aspirin circumvented the resistance of 4T1 cells in the co-culture system to GDC-0941, leading to significantly reduced cell proliferation (Fig. 4a). Consistently, in contrast to single-agent treatments, combined use of GDC-0941 and Aspirin led to significantly increased apoptotic cell death in 4T1 cells co-cultured with macrophages (Fig. 4b). Immunofluorescent staining analysis revealed that 4T1 cells co-cultured with RAW264.7 macrophages displayed cytoplasmic retention of p65 in response to Aspirin monotherapy or its combined use with GDC-0941 (Fig. 4c), suggesting a suppressive effect of Aspirin on NF-κB activation. It has been previously demonstrated that phosphorylation of IκBα releases NF-κB, permitting its activation and translocation into the nucleus^25^. Our western blot analysis revealed that GDC-0941 treatment resulted in a substantial increase in phosphorylated IκBα whereas additional use of Aspirin treatment abrogated this signal in 4T1 cells co-cultured with macrophages (Supplementary Fig. S3). Together, these results suggested that Aspirin may inhibit NF-κB signaling, sensitizing 4T1 cells to PI3K inhibition by GDC-0941 in the presence of macrophages in the microenvironment.

### Combined use of Aspirin and GDC-0941 effectively suppressed tumor growth and lung metastasis

**Fig. 5:**
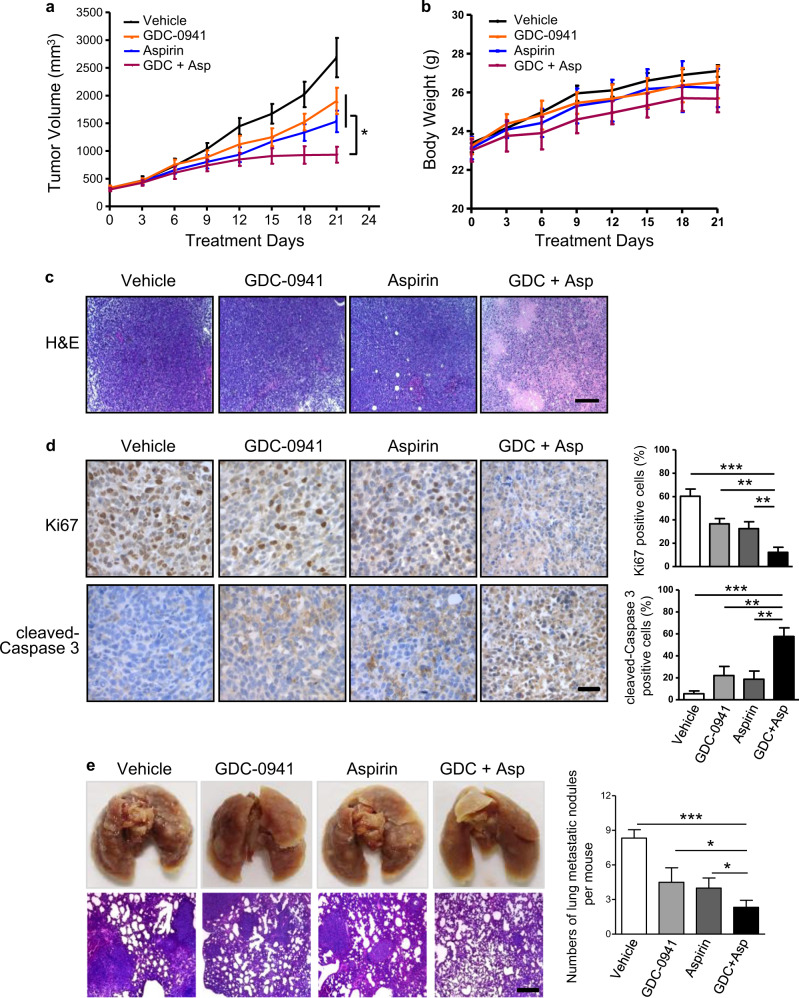
Aspirin and GDC-0941 cooperated to suppress tumor growth and lung metastasis in vivo. **a** Tumor volume changes over time in mice bearing 4T1 allografts following treatment with GDC-0941 (125 mg/kg/day, oral gavage) and Aspirin (100 mg/kg/day, oral gavage) as single agents or in combination (*n* = 5–6 per treatment group). Error bars represent mean ± S.E.M. **b** Changes in body weight of 4T1 tumor-bearing mice during 21 days treatments with GDC-0941 (125 mg/kg/day, oral gavage) and Aspirin (100 mg/kg/day, oral gavage) as single agents or in combination (*n* = 5–6 per treatment group). Error bars represent mean ± S.E.M. **c** Representative images of histological staining for 4T1 tumors from Balb/c mice (*n* = 5 per treatment group) following treatment with GDC-0941 and Aspirin as single agents or in combination for 4 days. Scale bar, 200 μm. **d** Representative images of immunohistochemical staining for proteins as indicated in 4T1 tumors from Balb/c mice (*n* = 5 per treatment group) following treatment with GDC-0941 and Aspirin as single agents or in combination for 4 days. Scale bar, 50 μm. Data are shown as mean ± S.E.M. **e** Representative images of gross examination (upper panel) and histological staining (lower panel) of lung metastasis in mice bearing 4T1 tumors treated with GDC-0941 and Aspirin as single agents or in combination (*n* = 5 per treatment group) for 21 days. Scale bar, 200 μm. The numbers of lung metastatic nodules per mouse are shown as mean ± S.E.M. *P < 0.05; **P < 0.01; ***P < 0.001 (Student’s *t*-test)

We next assessed the effect of the drug combination on mice bearing 4T1 allograft tumors. Once tumors were established in Balb/c mice, we treated them with GDC-0941 (125 mg/kg) and Aspirin (100 mg/kg) as single agents or in combination. While GDC-0941 monotherapy moderately attenuated tumor growth, combined use of GDC-0941 and Aspirin effectively slowed down the tumor growth, leading to stable disease (Fig. 5a). We observed no overt toxicity as none of the treatments caused significant weight loss in the tumor-bearing mice examined (Fig. 5b). Strikingly, we observed substantially large areas of necrosis only in the combination drug-treated tumors (Fig. 5c). Further immunohistochemical analysis revealed that in contrast to the vehicle or single-agent-treated group, the tumors treated with combined GDC-0941/Aspirin, specifically in the non-necrotic tumor area, exhibited significantly reduced proliferation as determined by Ki67 staining positive cells and increased apoptosis as determined by cleaved-Caspase 3-positive cells (Fig. 5d). Thus, decreased proliferation and increased apoptosis may explain, at least in part, the effectiveness of the combination treatment. Notably, mice treated with GDC-0941 in combination with Aspirin showed a drastic reduction in lung metastatic nodules (Fig. 5e), indicating the combination treatment may synergize to block metastasis of breast cancer cells.

**Fig. 6:**
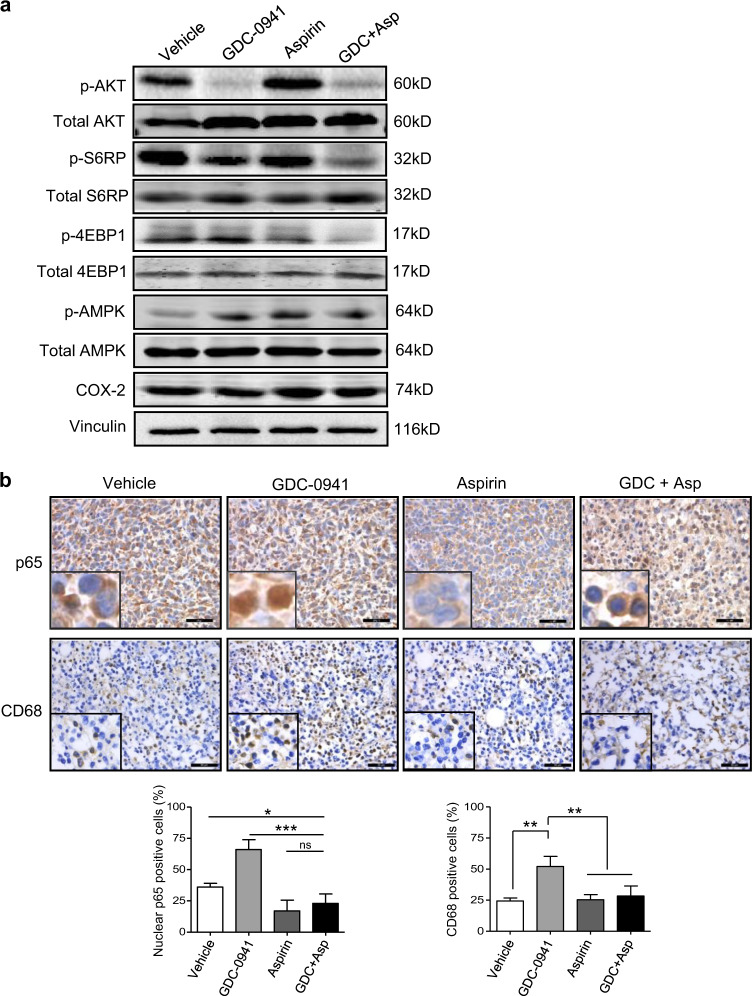
Combined use of GDC-0941 and Aspirin significantly inhibited the NF-κB signaling and macrophages infiltration in vivo. **a** Western blot analysis of proteins as indicated in 4T1 tumors of Balb/c mice following treatment with GDC-0941 (125 mg/kg/day, oral gavage) and Aspirin (100 mg/kg/day, oral gavage) as single agents or in combination (*n* = 5 per treatment group) for 3 days. Vinculin was used as a loading control. **b** Representative images of immunohistochemical staining for p65 and CD68 of 4T1 tumors after the mice were treated with GDC-0941 and Aspirin as single agents or in combination (*n* = 5 per treatment group) for 3 and 10 days, respectively. Scale bar, 50 μm. Percentages of CD68 staining positive cells and nuclear p65 staining positive cells are shown. Error bars represent mean ± S.E.M. *P < 0.05; **P < 0.01; ***P < 0.001 (Student’s *t*-test)

The growth inhibitory effect of Aspirin has been ascribed to AMPK activation and consequent inhibition of mTOR signaling^26,27^, or COX-2 inactivation^28,29^. Our western blot analysis revealed that Aspirin as a single agent or in combination with GDC-0941 resulted in markedly increased levels of phosphorylated AMPK but had little impact on COX-2 expression (Fig. 6a). Consistent with a role of AMPK activation in negative regulation of mTOR signaling^30^, Aspirin treatment also led to markedly reduced p4EBP1 and modestly reduced pS6RP levels, whereas combined use of Aspirin and GDC-0941 resulted in nearly diminished mTOR signaling (Fig. 6a).Together, these results suggested that Aspirin may suppress tumor growth by activation of AMPK and inhibition of mTOR signaling, but less likely by inhibiting COX-2.

Several lines of evidence suggest that Aspirin has potent inhibitory effect on NF-κB-mediated inflammation and macrophage infiltration^21,22,31,32^. Immunohistochemical staining analysis revealed that while GDC-0941 induced nuclear localization of p65 in 4T1 tumors cells, Aspirin monotherapy or in combination with GDC-0941 resulted in substantial increase in cytoplasmic localization of p65 (Fig. 6b), suggesting a suppressing effect of Aspirin on NF-κB activation induced by PI3K inhibition. We also observed a pronounced reduction in CD68 staining in the 4T1 tumors from mice treated with Aspirin monotherapy or in combination with GDC-0941 (Fig. 6b), suggesting that macrophage infiltration induced by PI3K inhibition can be blocked by Aspirin administration. Together, combined use of GDC-0941 and Aspirin demonstrated significant anti-tumor activity in the 4T1 allograft model of breast cancer.

## Discussion

As PI3K signaling is frequently deregulated in breast cancer, targeting this pathway poses a promising therapeutic strategy^1^. However, recent clinical trials reveal that PI3K inhibitor monotherapy exhibits only modest anti-tumor activity, and often leads to the development of resistance and relapse of the disease^2,3^. To search for potential resistance mechanisms, we employed a 4T1 mouse breast tumor model in which murine epithelial cancer cells grow in their natural microenvironment. Unlike its strong growth inhibitory effect in vitro, PI3K inhibition by GDC-0941 only modestly attenuated tumor growth. Interestingly, we noticed pronounced infiltration of TAMs and induction of the expression of several macrophage-associated cytokines and chemokines in drug-treated tumors. We further found that macrophages conferred resistance to PI3K inhibition through the activation of NF-κB signaling in 4T1 cells. Moreover, we showed that Aspirin could block the activation of macrophage-mediated NF-κB signaling induced by PI3K inhibition, and combined use of GDC-0941 and Aspirin significantly reduced tumor burden, macrophage infiltration, and pulmonary metastasis in the 4T1 mouse breast tumor model.

The transcription factor NF-κB is a common mediator of tumorigenesis, tumor development, and drug resistance^19^. NF-κB-dependent IL-6 production and the downstream STAT3 signaling have been demonstrated to promote neoplastic cell proliferation and survival, as well as induce chemoresistance of diverse tumor cell lines in vitro^33,34^. A recent study reported that TNFα produced by macrophages rendered melanoma cells resistant to MEK inhibition through activation of NF-κB signaling^20^. In the current study, we found that blockade of PI3K signaling induced activation of the NF-κB pathway in 4T1 cells when co-cultured with macrophages, indicating a potential mechanism of resistance to PI3K inhibition. While these findings supported a potential role of macrophages in the activation of the NF-κB signaling pathway by 4T1 cells, the underlying mechanism remains to be investigated.

Growing evidence supported a role of the anti-inflammatory agent Aspirin in cancer prevention^29,35^. Aspirin has been shown to inhibit the activation of the NF-κB pathway and block the growth of cancer cells^21–24^. In the current study, we show that Aspirin suppresses NF-κB activation and macrophage infiltration that are induced by GDC-0941 treatment. We also provide evidence that Aspirin may suppress tumor growth by activation of AMPK and inhibition of mTOR signaling. However, the exact impact of PI3K and Aspirin on the activity of the individual immune-cell populations within the tumor microenvironment remains to be investigated in future studies.

To date, a large number of clinical trials with Aspirin or PI3K inhibitors as monotherapies or each in combination with other agents are underway (www.clinical trials.gov). A recent study reported that combination therapy of Aspirin and PI3K inhibitors significantly suppressed human breast cancer in epithelial cell monoculture and xenograft models of immunodeficient mice^27^. In contrast, our study investigated the effect of such drug combination using 4T1 mouse breast tumor epithelial cells in the co-culture system with macrophages as well as the immunocompetent mouse tumor model in which epithelial cancer cells are surrounded by their own natural microenvironment with intact immune components, more mimicking the disease situation in human. Together, the current study identifies the tumor microenvironment as a likely source of resistance to PI3K pathway inhibitors through macrophage-associated cytokines/chemokines. Furthermore, we find that Aspirin may enhance the efficacy of PI3K pathway inhibitors through suppressing NF-κB activation and macrophage infiltration. Our study thus provides a rationale for the combined use of Aspirin and PI3K inhibitors, and warrants clinical evaluation of this combination strategy in breast cancer treatment.

## Materials and methods

### Cell culture and reagents

4T1 and RAW264.7 cell lines were purchased from American Type Culture Collection and cultured in Dulbecco's modified Eagle's medium (DMEM) with 10% fetal bovine serum (FBS), 100 U/ml penicillin and streptomycin. Both cell lines were kept in culture for no more than 3 months after resuscitation, and tested periodically for Mycoplasma infection. GDC-0941 and Aspirin were purchased from Selleckchem (Houston, TX, USA) and Sigma-Aldrich (St Louis, MO, USA), respectively. For in vitro studies both compounds were diluted in dimethyl sulfoxide (DMSO). For in vivo use GDC-0941 was freshly formulated in 0.5% methyl cellulose/Tween 80 and Aspirin was freshly formulated in 0.5% sodium-carboxymethyl cellulose/Tween 80.

### Clonogenic proliferation assay

Cell proliferation was measured as previously described^20^. 4T1 cells were seeded into 12-well plates at 10,000 cells/well density and then treated with GDC-0941 and Aspirin as single agents or in combination. Fresh media were replaced every 2 days until untreated cells reached confluence. 4T1 cells were then fixed with 4% formaldehyde, and stained with crystal violet. Cells were imaged with Molecular Imager (Bio-Rad Laboratories, Hercules, CA, USA). For quantification of the relative cell number, 10% acetic acid was added to each well to extract the dye and the absorbance was measured at 570 nm.

### Transwell co-culture assay

4T1 cells were co-cultured with either RAW264.7 macrophages or primary macrophages derived from mouse bone marrow (BMM) without cell to cell contact. Briefly, 5 × 10^3^ macrophages were seeded in transwell inserts (0.4 μm pores; Corning Costar, Corning, NY, USA), consisting of a membrane permeable for liquids but not cells. The macrophages were pre-incubated for 48 h with 4T1-conditioned medium to activate macrophages. The inserts were placed in a 12-well plate with pre-seeded 4T1 cells. Experiments using drugs were performed for 24 h by adding GDC-0941 and Aspirin as single agents or in combination to the wells, so that both cell populations were exposed to the same conditions.

### Apoptosis assay

Following 48 h drug treatment, 4T1 cells in in vitro co-culture system were stained with Annexin V FITC and PI solution according to the manufacturer’s protocol (Dojindo Molecular Technologies, Tabaru, Japan). Cell apoptosis was quantified by the flow cytometry analysis on a BD Accuri™ C6 (BD Biosciences, San Jose, CA, USA).

### Mouse bone marrow monocytes isolation and differentiation

Mouse BMMs were obtained from femurs of about 8-week-old BALB/c mice as stated previously^36^. Briefly, bone marrow cavities were flushed with phosphate-buffered saline. After centrifugation at 500 *g* for 10 min, bone marrow cells were cultured for 7 days in DMEM complete medium supplemented with recombinant human CSF1 (10^4^ U/ml; Sigma-Aldrich) on a 10 cm Petri dish.

### In vitro migration assay

5 × 10^4^ RAW264.7 or BMM macrophages were seeded in the transwell inserts (8 μm pores, Corning Costar) in serum-free medium. The inserts were then placed in a 24-well plate with conditioned media from 4T1 cells pre-treated with GDC-0941 or DMSO vehicle. The cells were kept in culture for 24 h at 37 °C and then the migrated cells were stained with 0.5% crystal violet and the number of migrated cells was scored by an inverted phase contrast microscope (Leica Microsystems, Wetzlar, Germany). Migrated cells were quantified using Image J software.

### Western blot and antibodies

Cell lysates were prepared using RIPA buffer (Sigma-Aldrich) supplemented with proteinase and phosphatase inhibitors (Roche, Indianapolis, IN, USA). Proteins were quantitated using BCA kit (Transgene, Beijing, China), separated by SDS-PAGE, and blotted onto nitrocellulose membranes. Proteins were probed with the following antibodies: p-AKT (Ser473; Cell Signaling Technology, Beverly, MA, USA; 4060), AKT (CST 2920), p-S6RP (Ser235/236, CST 4858), S6RP (Proteintech, Rosemont, IL, USA; 14823-1-AP), p-4EBP1 (Thr37/46, CST 2855), 4EBP1 (Proteintech 60246-1-AP), p-NF-κB p65 (Ser536, CST 3033), NF-κB p65 (Proteintech 10745-1-AP), p-IκBα (Ser32, CST 2859), IκBα (CST 9242), p-AMPK (Thr172, CST 50081), AMPK (CST 5831), COX-2 (CST 12282), and Vinculin (Sigma-Aldrich V9131). Immunofluorescently labeled secondary antibodies to mouse IgG (Rockland Immunochemicals, Limerick, PA, USA) or rabbit IgG (Molecular Probes, Grand Island, NY, USA) were used to visualize blots on an Odyssey scanner (Li-Cor, Lincoln, NE, USA).

### ELISA assay

4T1 cells and RAW264.7 cells were mixed at a 3:1 cell number ratio. After 24 h of co-culture, the mixed cells were treated with DMSO or GDC-0941 for another 24 h. Protein concentrations of TNFα and IL-6 in culture supernatants were measured by ELISA assay using a TNFα ELISA kit (Biolegend, San Diego, CA, USA) and an IL-6 ELISA kit (R&D Systems, Minneapolis, MN, USA) in accordance with the manufacturers’ instructions.

### RNA isolation and quantitative RT-PCR analysis

RNA from cell lines was isolated with TRizol (Life Technologies, Grand Island, NY, USA). RNA from frozen sections of mouse tumor kept in TRizol for 2 h was similarly isolated. Reverse transcription reaction was conducted using PrimeScript™ RT reagent Kit (Takara, Kusatsu, Japan). Quantitative RT-PCR was performed using SYBR Green PCR master Mix (Life Technologies) on a real-time quantitative PCR detection system (Agilent technologies, Santa Clara, CA, USA). Primers used in the real-time qPCR analysis are shown in Supplementary Table 1.

### Immunofluorescence assay

Immunofluorescence assay was carried out in in vitro co-culture system to detect cellular localization of the NF-κB p65 subunit in 4T1 cells. For this, 4T1 cells were seeded on glass coverslips and co-cultured with RAW264.7 macrophages in the presence of drugs as indicated in the figure legends. After drug treatments, 4T1 cells were fixed in 4% paraformaldehyde, permeabilized with 0.1% Triton X-100, and blocked with 5% bovine serum albumin. Cells were then incubated with rabbit anti-p65 antibody (Proteintech 10745-1-AP) overnight at 4 °C. Cells were then incubated with fluorescence-conjugated secondary antibody and DAPI at room temperature. Images were acquired and quantified using a upright fluorescence microscope (Leica Microsystems).

### Animal experiments

All animal experiments were carried out in strict accordance with protocols approved by Animal Research Committee of Dalian Medical University. Female Balb/c mice were purchased from SPF Animal Experiment Centre of Dalian Medical University. To establish 4T1 allografts, 5 × 10^5^ cells were injected into mammary fat pad of 8-week-old BALB/c mice, and the mice were monitored routinely for tumor growth. The tumors volumes were allowed to reach at about 300 mm^3^. Mice with similar tumor volumes were randomized into control and treatment groups, and five or six mice were used for each experimental cohort. For drug treatment, mice were dosed daily by oral gavage with vehicle, GDC-0941 (125 mg/kg), Aspirin (100 mg/kg) or GDC-0941 (125 mg/kg) + Aspirin (100 mg/kg) for the indicated time. Tumor size was measured every 3 days and at the end point using digital calipers and tumor volume was calculated by the formula: 0.5 × (length [mm]) × (width [mm])^2^.

### Cytokine array

The Proteome Profiler Mouse Cytokine Array kit (R&D systems) was utilized according to the manufacturer’s instructions. Briefly, excised tumors were homogenized in RIPA buffer with protease inhibitors, and 250 μg of lysates were incubated with blocked membranes overnight. Membranes were then washed and exposed to chemiluminescent reagent. Blot images were captured by ChemiDoc XRS + system (Bio-Rad, Hercules, CA, USA) and analyzed using Image J software.

### Histology and immunohistochemistry

Tumors and lungs were fixed in formalin overnight before paraffin embedding. Paraffin blocks were sectioned and stained with hematoxylin and eosin (H&E) for tumor histological and metastatic assessment. Immunohistochemistry (IHC) was carried out as described previously^7^. The following antibodies were used: p-AKT (Ser473; Invitrogen, Carlsbad, CA, USA; 785697A), CD68 (Abcam, Cambridge, MA, USA; ab125212), NF-κB p65 (Proteintech 10745-1-AP), Ki67 (Abcam ab15580), cleaved-Caspase 3 (CST Asp175). Images were acquired and quantified using a upright optical microscope (Leica Microsystems). For each tumor sample, five random 40X fields were scored. The expression level of p-AKT was quantified by integrated optical density (IOD) with Image Pro Plus software. The percentage of CD68 and cleaved-Caspase 3 staining positive cells, and nuclear Ki67 and p65 staining positive cells was calculated.

### Statistical analysis

If not indicated otherwise, each experiment was repeated independently at least three times. Quantitative results were analyzed by two-tailed unpaired Student’s *t*-test. A *P-*value of <0.05 was considered statistically significant.

## Electronic supplementary material

Supplementary Figures and Table
