# Author Correction: Macrophages confer resistance to PI3K inhibitor GDC-0941 in breast cancer through the activation of NF-κB signaling

**DOI:** 10.1038/s41419-019-1692-0

**Published:** 2019-06-24

**Authors:** Muhammad Waqas Usman, Jing Gao, Tiezheng Zheng, Chunhua Rui, Ting Li, Xing Bian, Hailing Cheng, Pixu Liu, Fuwen Luo

**Affiliations:** 1 Cancer Institute, Department of Acute Abdomen Surgery, The Second Hospital of Dalian Medical University, Institute of Cancer Stem Cell, Dalian Medical University, Dalian, 116044 China; 2 Department of Physiology, Institute of Basic Medical Sciences, Dalian Medical University, Dalian, 116044 China; 3 College of Pharmacy, Dalian Medical University, Dalian, 116044 China

**Correction to:** ***Cell Death & Disease***

10.1038/s41419-018-0849-6, published online 24 July 2018

Since publication of this article, the authors have noticed the following errors:

1. Figure 3c, the image is correct but the authors mistakenly provided incorrect figure legend. The correct figure legend is included below along with the original figure.

**Figure:**
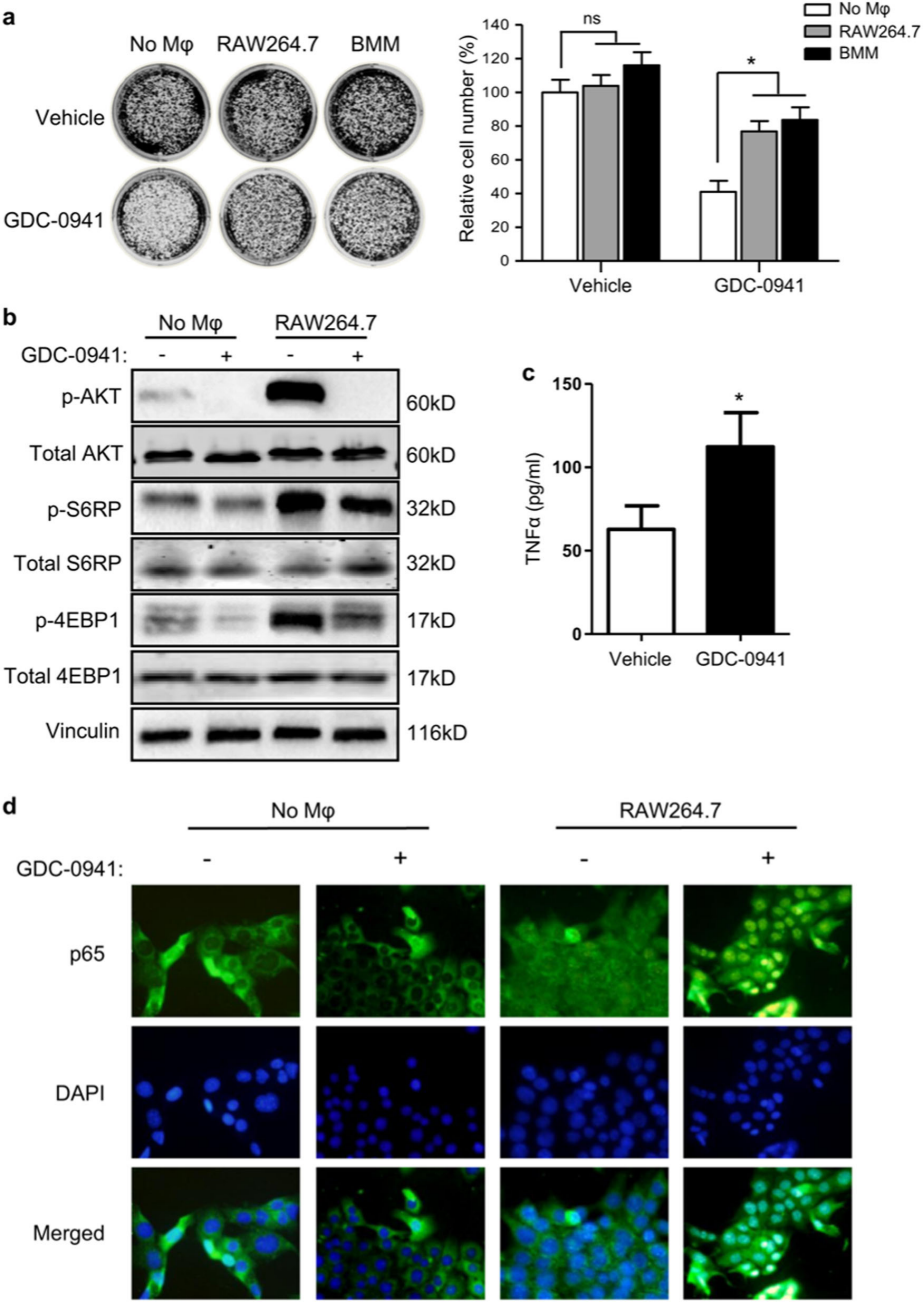
Fig. 3
2. Supplementary Fig. S2, the authors mistakenly provided the data from ELISA analysis of TNFα and IL-6 in media from co-cultured 4T1 and RAW264.7 cells. As stated in the main text, data from ELISA analysis of TNFα and IL-6 in 4T1 tumors from Balb/c mice treated with GDC-0941 should be provided. The correct figure and figure legend are included below.
3. The authors noticed an error in the manuscript in which “RAW276.7” should be “RAW264.7”.

The corrections do not alter the conclusions of the paper. The authors apologize for any inconvenience caused.

This has been corrected in both the PDF and HTML versions of the Article.

