# Supplementary material for: Macrophages confer resistance to PI3K inhibitor GDC-0941 in breast cancer through the activation of NF-κB signaling
Source: Cell Death Dis. 2018 Jul 24;9(8):809. doi: 10.1038/s41419-018-0849-6 (PMC6057974; doi:10.1038/s41419-018-0849-6)
Supplement: Supplementary file 1 — Supplementary Figures and Table [file 41419_2018_849_MOESM1_ESM.pdf]

Supplementary Figure S1

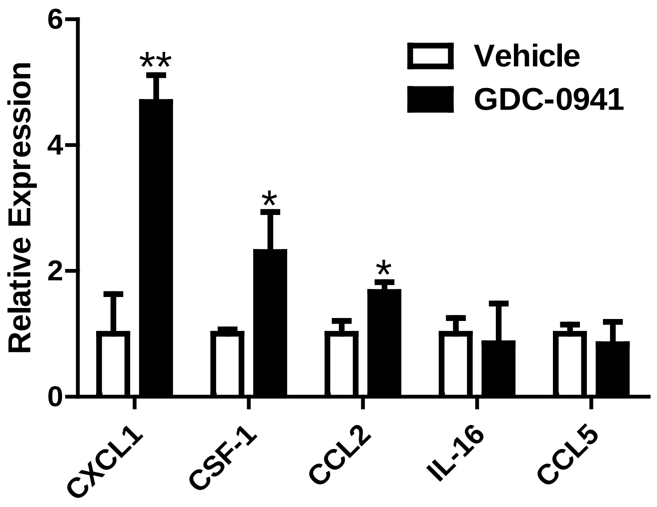

Supplementary Figure S1. Cytokine Array analysis. Listed is the relative fold induction of protein expression of CXCL1, CSF-1, CCL2 and CCL5 in 4T1 tumors from Balb/c mice treated with GDC-0941 (n=5). The vehicle-treated group (n=5) was used as a control. Protein abundance was calculated by densitometry from immunoblots. Error bars represent mean ± S.E.M. *$P< 0.05$; **$P< 0.01$ (Student's *t* test).

Supplementary Figure S2

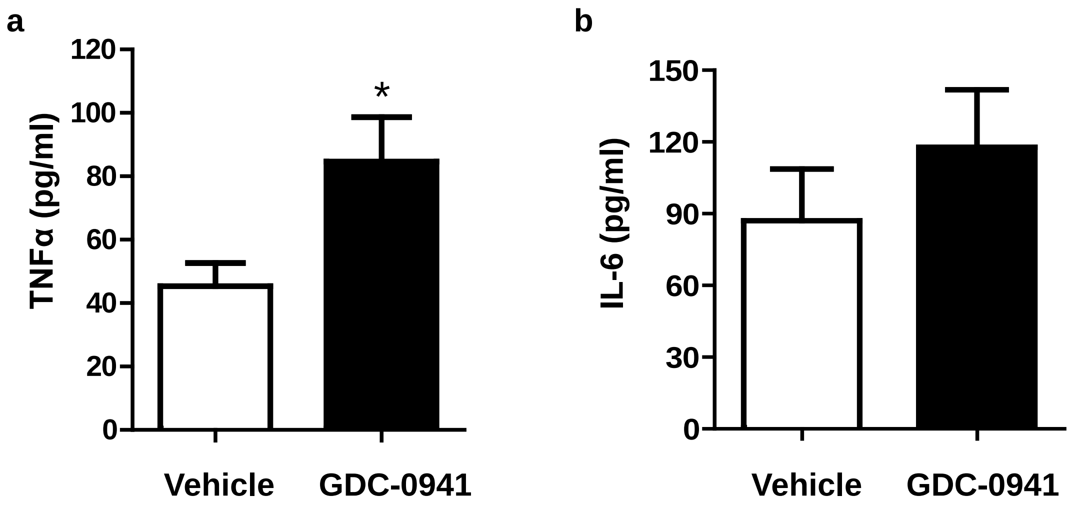

Supplementary Figure S2. ELISA analysis. Production of TNFα (a) and IL-6 (b) in 4T1 tumors from Balb/c mice treated with GDC-0941 (n=5). The vehicle-treated group (n=5) was used as a control. Error bars represent mean ± S.E.M. *P< 0.05 (Student's *t* test).

Supplementary Figure S3

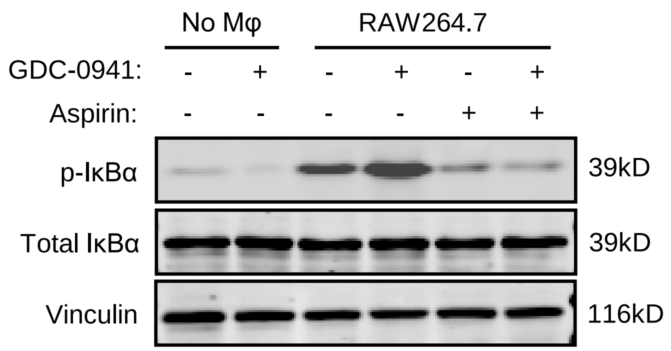

Supplementary Figure S3. Western blot analysis of p-IκBα and total IκBα in 4T1 monoculture or co-cultured with RAW276.7 macrophages in the presence or absence of GDC-0941 and/or Aspirin. Vinculin was used as a loading control.

Supplementary Table 1

Primer Name	Primer Sequence
TNFα-F	CCTTGTTGCCTCCTCTTTTGC
TNFα-R	TCAGTGATGTAGCGACAGCCTG
IL6-F	TAGTCCTTCCTACCCCAATTTCC
IL6-R	TTGGTCCTTAGCCACTCCTTC
CXCL1-F	ACTGCACCCAAACCGAAGTC
CXCL1-R	TGGGGACACCTTTTAGCATCTT
CSF1-F	ATGAGCAGGAGTATTGCCAAGG
CSF1-R	TCCATTCCCAATCATGTGGCTA
CCL2-F	TTAAAAACCTGGATCGGAACCAA
CCL2-R	GCATTAGCTTCAGATTTACGGGT
CCL5-F	GCTGCTTTGCCTACCTCTCC
CCL5-R	TCGAGTGACAAACACGACTGC
IL16-F	AAGAGCCGGAAATCCACGAAA
IL16-R	GTCTCAAAAGGGTCAGGGTACT
IL17-F	TTTAACTCCCTTGGCGCAAAA
IL17-R	CTTTCCCTCCGCATTGACAC

Supplementary Table 1. List of primers and their sequences used in this study.
